# A multifaceted comparative analysis of image and video technologies in gastrointestinal endoscope and their clinical applications

**DOI:** 10.3389/fmed.2023.1226748

**Published:** 2023-10-10

**Authors:** Yuequn Chen, Guiqiong Wu, Chaojun Qu, Zimao Ye, Yihao Kang, Xin Tian

**Affiliations:** Department of Intensive Care Unit, Fifth Affiliated Hospital of Wenzhou Medical University, Lishui Municipal Central Hospital, Lishui, China

**Keywords:** gastrointestinal disease diagnosis, endoscopy-guided Nasojejunal feeding tube placement, image and video, real-time monitoring, enteral nutrition

## Abstract

This paper presents a comprehensive exploration of endoscopic technologies in clinical applications across seven tables, each focusing on a unique facet of the medical field. The discourse begins with a detailed analysis of pediatric endoscopes, highlighting their diagnostic capabilities in various conditions. It then delves into the specifications and applications of globally recognized capsule endoscopy devices. Additionally, the paper incorporates an analysis of advanced imaging techniques, such as Narrow Band Imaging (NBI), Flexible Spectral Imaging Color Enhancement (FICE), and i-scan, which are increasingly being integrated into ultrathin gastrointestinal (GI) endoscopes. Factors like technological capabilities, light source, camera technology, and computational constraints are evaluated to understand their compatibility with these advanced imaging techniques, each offering unique advantages and challenges in clinical settings. NBI, for instance, is lauded for its user-friendly, real-time enhanced imaging capabilities, making it effective for early detection of conditions like colorectal cancer and Barrett’s esophagus. Conversely, FICE and i-scan offer high customizability and are compatible with a broader range of endoscope models. The paper further delves into innovative advances in movement control for Nasojejunal (NJ) feeding tube endoscopy, elucidating the potential of AI and other novel strategies. A review of the technologies and methodologies enhancing endoscopic procedure control and diagnostic precision follows, emphasizing image and video technologies in pediatric endoscopy, capsule endoscopes, ultrathin endoscopes, and their clinical applications. Finally, a comparative analysis of leading real-time video monitoring endoscopes in clinical practices underscores the continuous advancements in the field of endoscopy, ensuring improved diagnostics and precision in surgical procedures. Collectively, the comparative analysis presented in this paper highlights the remarkable diversity and continuous evolution of endoscopic technologies, underlining their crucial role in diagnosing and treating an array of medical conditions, thereby fostering advancements in patient care and clinical outcomes.

## Introduction

Feeding tube nutrition is critically important for ICU (intensive care unit) patients who are unable to eat or digest food normally due to illness, injury, or surgery (1). ICU patients are often critically ill and require specialized nutritional support to help them recover and maintain their health. Feeding tube nutrition can provide essential nutrients that ICU patients need to maintain their health, such as proteins, carbohydrates, fats, vitamins, and minerals (2). Feeding tube nutrition improves patients’ mental health and inflammation (3). The practice can reduce nutritional morbidity and hospital length of stay (4). This is particularly important for patients who are unable to eat or digest food normally, as they may not be getting the nutrients they need from their normal diet. ICU patients are often vulnerable to infections and other complications due to their weakened immune systems (5). Feeding tube nutrition can help support immune function by providing nutrients that are essential for the growth and function of immune cells (6, 7). ICU patients are at risk of losing muscle mass due to their illness, injury, or prolonged bed rest. Feeding tube nutrition can help maintain muscle mass by providing essential amino acids and other nutrients that are necessary for muscle growth and repair (8, 9). Many ICU patients have wounds that require healing, such as surgical incisions or bedsores. Feeding tube nutrition can provide the nutrients that are necessary for wound healing (8). ICU patients are at risk of developing complications related to their illness or injury, such as infections, pressure ulcers, and organ failure (10, 11). Feeding tube nutrition can help reduce the risk of these complications by providing the nutrients that are necessary for the body to function properly.

Nasojejunal (NJ) feeding tubes and nasogastric (NG) feeding tubes are both types of feeding tubes that are used to provide nutritional support to patients who are unable to eat or digest food normally (12–14). NJ feeding tubes are inserted through the nose and passed through the stomach into the small intestine, where they deliver nutrition directly to the jejunum (the middle section of the small intestine) (15). NJ feeding tubes are often used in patients who have impaired gastric function (16) or who are at risk of aspiration (breathing in food or liquid into the lungs) (17), such as critically ill patients or those who have undergone certain types of surgery (18). In contrast, nasogastric feeding tubes are inserted through the nose and passed through the esophagus into the stomach, where they deliver nutrition directly to the stomach (19). NG feeding tubes are often used in patients who have a functioning gastrointestinal tract but are unable to eat or swallow normally, such as those with neurological or muscular disorders (20). NJ tubes may be associated with a higher risk of complications such as dislodgement (21), migration (22), or bowel perforation (23), while nasogastric tubes may be associated with a higher risk of aspiration (24) or reflux (25). NJ feeding tubes may be preferred in ICU patients who have impaired gastric function or are at risk of aspiration, as they allow for direct delivery of nutrition to the small intestine and bypass the stomach (26). This can reduce the risk of aspiration and may be beneficial in patients with severe respiratory compromise or gastrointestinal dysfunction.

There have been several recent advances in NJ feeding tube technology that have improved their safety, comfort, and effectiveness: developments in tube materials and manufacturing have led to the development of smaller and more flexible NJ feeding tubes (27, 28). This allows for easier insertion and greater comfort for the patient, as well as a reduced risk of injury to the nasal and intestinal tissues. Some NJ feeding tubes now feature radiopaque markers that can be seen on X-rays or other imaging tests (29). This makes it easier for healthcare providers to confirm proper placement of the tube and ensure that the tube is positioned in the jejunum. However, radiopaque markers require the use of imaging techniques such as X-rays, CT scans, or fluoroscopy to confirm placement, which can expose patients to potentially harmful levels of radiation (30). Radiopaque markers can make the feeding tube stiffer and less pliable (31), making it more difficult to pass through the nasal passage and into the jejunum. The addition of radiopaque markers can increase the weight and rigidity of the feeding tube, increasing the risk of dislodgement and requiring more frequent repositioning. In some cases, radiopaque markers may not be clearly visible on imaging studies, making it difficult to determine proper placement.

Integrated jejunal extension of NJ feeding tubes (IJENJ) is a technique used to provide enteral feeding to patients who cannot tolerate oral or gastric feeding (32, 33). While IJENJ can be an effective method for providing enteral nutrition, there are several disadvantages to this technique that should be considered. The placement of an IJENJ tube requires a high level of skill and experience (34), and may not be possible in all patients. Additionally, the tube may become dislodged or obstructed (35), requiring intervention to reposition or replace it. Any invasive medical procedure carries a risk of infection, and the placement of an IJENJ tube is no exception. Patients with IJENJ tubes are at risk of developing infections at the insertion site, or in the small intestine if the tube becomes contaminated (36). Patients may experience discomfort or pain during the placement of an IJENJ tube, or during the period of time when the tube is in place. Additionally, patients with IJENJ tubes may have limited mobility or activity levels due to the presence of the tube (37).

Magnetic tip guidance is a technique used during the placement of NJ feeding tubes (38, 39). This technique involves the use of a magnetic-tipped guidewire to guide the feeding tube through the gastrointestinal tract and into the small intestine. Although this technique has some advantages over other methods, there are also some disadvantages that should be considered. Magnetic tip guidance requires the insertion of a guidewire through the nose or mouth and into the gastrointestinal tract. This procedure can increase the risk of perforation, especially if the guidewire is not properly positioned or if there are pre-existing conditions that increase the risk of injury (40). Magnetic tip guidance is not suitable for all patients, as it requires the use of magnetic imaging equipment that may not be available in all hospitals or medical facilities. Magnetic tip guidance can be more expensive than other methods of feeding tube placement, as it requires the use of specialized equipment and trained personnel. The presence of other magnetic objects in the vicinity of the patient, such as medical equipment or jewelry, can interfere with the magnetic tip guidance process and lead to inaccurate placement of the feeding tube (41). The use of a guidewire increases the risk of infection, as it can introduce bacteria or other pathogens into the gastrointestinal tract (42).

Bedside ultrasound is a useful tool for verifying the placement of NJ feeding tubes (43). However, there are some potential disadvantages to using this method. The accuracy of bedside ultrasound in verifying the placement of NJ feeding tubes depends on the experience and skill of the operator. Inexperienced operators may have difficulty identifying the tube tip, which can lead to misinterpretation of the ultrasound images and incorrect placement verification (44). The use of bedside ultrasound may be limited in patients with anatomical variations or obesity, which can make it difficult to visualize the tube tip. Additionally, gastric contents or air in the gastrointestinal tract can interfere with the ultrasound image and make it harder to confirm the tube placement (45). Bedside ultrasound may not be able to detect certain complications associated with NJ feeding tube placement, such as misplacement in the tracheobronchial tree or intravascular migration. In these cases, additional imaging modalities, such as X-ray or computed tomography (CT), may be necessary for accurate verification.

Endoscopy-guided NJ feeding tube placement is an advanced technique that involves using an endoscope to guide the feeding tube into the small intestine (17, 27). This technique is typically reserved for patients who have difficult or complicated anatomy, or who have had previous failed attempts at NJ tube placement. Recent developments in endoscope technology have led to the development of smaller and more flexible endoscopes. This allows for easier insertion and greater comfort for the patient, as well as a reduced risk of injury to the nasal and intestinal tissues. Modern endoscopes have cameras that can produce detailed images of the inside of the patient’s digestive tract (46). This can help the healthcare provider to visualize the precise location of the feeding tube and ensure that it is placed correctly. Wireless capsule endoscopy is a technique in which a small, swallowable capsule containing a camera and transmitter is used to visualize the small intestine (47). This can be a useful tool for confirming the location of the feeding tube in patients with difficult anatomy or who have had previous surgery that may have altered the anatomy. Some advanced endoscopy systems allow for real-time video monitoring of the procedure. This can help the healthcare provider to monitor the placement of the feeding tube and make adjustments as needed (48). These visual placement techniques can simplify the process of confirming NJ tube placement, reduce the need for imaging, and improve patient comfort and safety. Understanding the advances in endoscopy technology will make the endoscopy-guided NJ feeding tube placement technique safer, more effective, and more comfortable for patients.

### The development of small endoscopes

Small endoscopes have also played an important role in the development of endoscopy-guided NJ feeding tubes, which are used to provide enteral nutrition to patients who are unable to take food orally. The use of small endoscopes in the placement of NJ feeding tubes has several advantages over traditional methods. Small endoscopes can provide real-time visualization of the tube placement, allowing for more accurate and precise placement of the tube in the small intestine (49).

#### Image and video technologies in pediatric endoscopy and their clinical applications

A pediatric endoscope is a medical instrument used to examine and treat various conditions in children, especially in the gastrointestinal (GI) tract. It’s a flexible, thin, lighted tube with a camera at the tip that allows healthcare professionals to visualize internal body structures. Pediatric endoscopes are designed to be smaller and more delicate than adult endoscopes, to accommodate the size and anatomy of children. The diameter of a pediatric endoscope typically ranges from 3 to 6 mm (Table 1) to fit the smaller size of a child’s GI tract. Smaller diameters (around 4.5 mm) are suitable for infants and very young children. Pediatric endoscopes are usually used with saline solution or other appropriate solutions to expand the GI tract, providing a clear view of the internal structures and allowing the endoscope to move more smoothly. This also helps in reducing the risk of injury to the GI tract during the procedure. The view field degrees in pediatric endoscopes vary, with most offering a wide field of view of 130–140 degrees (Table 1). This allows for a comprehensive visualization of the GI tract and helps in identifying any abnormalities or issues that may be present.

**Table 1:** Comparative analysis of pediatric gastrointestinal endoscopes across diverse clinical applications.

Manufacturer (city, country)	Model	Imaging quality	Diameter	View field	Potential applications	Capacity of processors	NBI	FICE	i-scan	Advantages	Limitations
Smith and Nephew (London, UK)	560H	HD	5 mm	130°	Pediatric Endoscopy	Camera Control Unit, 560H 3-Chip HD Camera Head, 560H Coupler				Pediatric-friendly size	Limited field of view
Pentax (Tokyo, Japan)	EC-34-i10	HD	4 mm	170°	Colonoscopy	Frequency: 10-MHz, RAM: 4 MB			Yes	General purpose, versatile	Limited by 10 MHz frequency
Pentax (Tokyo, Japan)	EC-38-i10P	HD	3 mm	170°	Colonoscopy	Frequency: 10-MHz, RAM: 8 MB			Yes	Good for specific procedures	May have a learning curve
Fujifilm (Tokyo, Japan)	EC-530WL	HD	4 mm	170°	Colonoscopy	Frequency: 20-MHz, RAM: 8 MB, Data Width: 16 bits		Yes		High-frequency, good for detailed imaging	Limited by data width
Fujifilm (Tokyo, Japan)	EC-530XS	HD	3 mm	170°	Colonoscopy	Frequency: 20-MHz, RAM: 8 MB, Data Width: 16 bits		Yes		High data width for detailed data	Complexity may require additional training
Pentax (Tokyo, Japan)	EG-1690 K	HD	6 mm	145°	Gastroscopy	Frequency: 2-MHz, RAM: 8 MB			Yes	Low frequency can be beneficial for specific applications	Lower memory, lower frequency limitations
Pentax (Tokyo, Japan)	EG-29-i10	HD	6 mm	140°	Gastroscopy	Frequency: 12-MHz, RAM: 4 MB			Yes	Lower frequency suitable for broader applications	Lower memory
Fujifilm (Tokyo, Japan)	EG-530FP	HD	6 mm	140°	Gastroscopy	Frequency: 20-MHz, RAM: 8 MB		Yes		High-frequency, good for detailed imaging	May be expensive
Fujifilm (Tokyo, Japan)	EG-530NP	HD	6 mm	140°	Gastroscopy	Frequency: 12-MHz and 20-MHz, RAM: 8 MB		Yes		Dual frequency options	May require specialized training
STERIS (Mentor, USA)	FlexiSight	HD	3 mm	135°	Gastroscopy, Colonoscopy	Frequency: 4 MHz, RAM: 8 MB				Good for tight spaces, smaller diameter	Lower resolution
Olympus (Tokyo, Japan)	GIF-N180	HD	4 mm	140°	Gastroscopy	Frequency: 20-MHz, RAM: 4 MB	Yes			Suitable for narrow applications	Limited memory
Olympus (Tokyo, Japan)	GIF-XP190N	HD	6 mm	145°	Gastroscopy	Frequency: 20-MHz, RAM: 8 MB	Yes			High-frequency for detailed imaging	May be expensive
ConMed (Utica, USA)	IM8000	HD	3 mm	140°	Pediatric Endoscopy	Frequency: 20 MHz, RAM: 4 GB, 4 MB				Good balance between speed and resolution	May have higher power consumption
Olympus (Tokyo, Japan)	IMH-20	HD	3 mm	140°	Gastroscopy	Frequency: 20-MHz, RAM: 8 MB, Data Width: 16 bits	Yes			Good balance of frequency and data width	May have a learning curve
Mindray (Shenzhen, China)	ME-8	HD	4.5 mm	130°	Gastroscopy, Colonoscopy	Memory: 18 MB, Processor Speed: 20 MHz, Additional Features: Up to 300 × 300 dpi				Compact and portable	May lack advanced features
Olympus (Tokyo, Japan)	PCF-PH190	HD	6 mm	170°	Colonoscopy	Data Width: 32 bits, Frequency: 33 MHz, RAM: 8 MB	Yes			32-bit data width for rich data	May require specialized equipment
Boston Scientific (Marlborough, USA)	SpyGlass DS	HD	4 mm	120°	Cholangioscopy, Pancreatoscopy	Frequency: 20–30 MHz, RAM: 4 GB, 4 MB	Yes	Yes		High frequency for detailed imaging	Limited to specialized applications
Olympus (Tokyo, Japan)	system-EVIS X1	HD	6 mm	140°	Gastroscopy	Data Width: 16 bits, Frequency: 12 MHz, RAM: 32 MB	Yes			Advanced features for various procedures	Limited by 16-bit data width
Vimex Endoscopy (Gdynia, Poland)	VE-4000	HD	4 mm	130°	Gastroscopy, Colonoscopy	Frequency: 20-MHz, RAM: 8 MB				High frequency for detailed imaging	High power consumption
Cook Medical (Bloomington, USA)	Video Endoscope	HD	4 mm	130°	Gastroscopy, Colonoscopy	RAM: 32 MB, Frequency: 10 MHz				General-purpose, versatile	May lack specialty features
Aohua (Shanghai, China)	VME-1000	HD	3 mm	140°	Gastroscopy, Colonoscopy	Frequency: 8 MHz, Data Width: 32 bits, Data Bus Width: 16 bits	Yes			High-resolution imaging	Limited to certain types of procedures
XION (Berlin, Germany)	XION Eluxeo	4K	3 mm	145°	Gastroscopy, Colonoscopy	Frequency: 50/60 Hz, Additional Features: Full HD, 4 LED lights	Yes	Yes		Multi-LED illumination for better imaging	May be expensive

Endoscopy-guided feeding tube placement is a minimally invasive procedure to insert a feeding tube directly into the stomach or small intestine (50). Pediatric endoscopes can be highly useful in this procedure as they allow for accurate visualization and precise placement of the feeding tube. This is especially important in children, who have smaller GI tracts and are at a higher risk of complications during the procedure. Using a pediatric endoscope for endoscopy-guided feeding tube placement offers several advantages: The real-time visualization reduces the risk of injury to internal structures and helps ensure accurate placement of the feeding tube. The procedure requires only a small incision, resulting in less pain, faster recovery, and reduced scarring. The procedure can be performed in less time compared to other methods, such as surgical placement, which is especially important for children who may have difficulty tolerating prolonged procedures.

Pediatric endoscopes are used for various objectives, including a variety of diagnostic and therapeutic procedures in pediatric patients (51). These endoscopes can be used for endoscopy-guided NJ feeding tube placement (52). The safety and efficacy of pediatric endoscopy was evaluated in children under the age of 18, including upper endoscopy, colonoscopy, and flexible sigmoidoscopy. Another study included upper endoscopy, colonoscopy, and enteroscopy, showed that pediatric endoscopy was effective in the diagnosis and treatment of a variety of gastrointestinal diseases, including inflammatory bowel disease (53, 54), and celiac disease (55, 56). Other study evaluated the use of pediatric endoscopy in the placement of gastrostomy tubes in children and found that pediatric endoscopy was a safe and effective method for gastrostomy tube placement in children, with a low rate of complications and high success rate (57). Overall, the evidence suggests that pediatric endoscopy is a safe and effective method for the diagnosis and treatment of pediatric gastrointestinal diseases, and can be used for a variety of diagnostic and therapeutic procedures. Researchers evaluated the use of pediatric endoscopes for the placement of NJ feeding tubes in pediatric patients. The study found that the use of pediatric endoscopes was safe and effective for the placement of NJ feeding tubes in this population (58). The study found that pediatric endoscopy was safe and effective, with a low rate of complications and high diagnostic yield (54). Researchers evaluated the use of pediatric endoscopes for the placement of NJ feeding tubes in infant and children patients who require intestinal feeding and found that the use of pediatric endoscopes for the placement of NJ feeding tubes in this population, with a success rate of 100% (59). The use of pediatric endoscopes in endoscopy-guided NJ feeding tubes has been shown to be effective in improving the accuracy and precision of the procedure while reducing the risk of complications (59, 60).

In GI endoscopy, the role of image quality is pivotal for accurate diagnoses and effective treatment plans. High-Definition (HD) technology typically offers a resolution of 1,920 × 1,080 pixels and a 16:9 aspect ratio. This level of detail is often more than adequate for a wide array of GI endoscopic procedures, from basic screenings to more complex evaluations. Its data usage is also lower—often requiring speeds of around 5 Mbps for streaming—which allows for easier integration into most existing healthcare data systems. Additionally, HD technology is generally more affordable, making it a cost-effective choice for many healthcare facilities. 4K Ultra-High Definition (UHD), with its much higher resolution of 3,840 × 2,160 pixels and similar 16:9 aspect ratio, is becoming increasingly relevant for specialized and complex GI procedures. The enhanced detail—essentially four times the pixels of HD—allows for better differentiation of tissue types and can be especially valuable for detecting subtle or early-stage lesions. However, 4K’s larger data size requires higher data speeds, usually around 25 Mbps for streaming, and more robust hardware capabilities. These higher requirements translate to increased costs, not just for the endoscopy units themselves but also for the computational power and data storage necessary to support them. Both HD and 4K have distinct advantages and limitations in the realm of GI endoscopy. HD provides reliable, high-quality imaging with lower data requirements and is more budget-friendly. In contrast, 4K offers unparalleled image quality and detail, although at a higher operational cost and with more demanding hardware and data requirements. The choice between the two will depend on the specific clinical needs, and the cases of former usage are more than latter (Table 1).

#### For chromoendoscopy, the following information was added

The application of advanced imaging techniques such as Narrow Band Imaging (NBI), Flexible Spectral Imaging Color Enhancement (FICE), and i-scan in ultrathin gastrointestinal (GI) endoscopes is contingent upon the technological capabilities of the specific endoscope model and the manufacturer’s design. NBI uses optical filters to enhance the visibility of vascular structures. The implementation of NBI is dependent on the light source and camera technology integrated into the endoscope. Ultrathin endoscopes equipped with this technology can certainly utilize NBI. FICE is a computational technique that post-processes images to enhance tissue contrast. Like NBI, the feasibility of FICE in ultrathin endoscopes depends on the device’s computational capabilities, which may be limited by the endoscope’s small diameter. i-scan is another digital image enhancement technology. Its implementation would also depend on the computational capabilities of the endoscope. Given the miniaturization of computational components, it is conceivable that ultrathin endoscopes could be equipped with i-scan technology.

NBI, FICE, and i-Scan each offer unique advantages in endoscopic imaging. NBI is often considered more user-friendly and provides real-time enhanced images, making it useful for practitioners across different systems and levels of expertise. It’s especially known for offering better contrast for vascular structures, a feature critical for the early detection of dysplastic lesions and forms of cancer like colorectal cancer and Barrett’s esophagus. The technology is also standardized across multiple endoscope models and manufacturers, which adds to its appeal. Extensive studies have been conducted on NBI, contributing to a strong evidence base supporting its efficacy in various clinical situations.

On the other hand, FICE and i-Scan come with their own sets of advantages. These technologies offer high customizability with multiple adjustable settings to suit specific clinical needs. They utilize advanced computer algorithms for post-processing, which can improve the quality of the images obtained. Furthermore, both FICE and i-Scan can often be employed with a wider range of endoscope models, including those that might not be compatible with NBI technology. While some studies suggest that these technologies might offer improved imaging for specific indications, the efficacy in comparison to NBI is still an area of ongoing research. Therefore, the choice between NBI, FICE, and i-Scan largely depends on the specific clinical case, the equipment available, and the endoscopist’s familiarity and comfort with the technology.

In the 2008 study by Yovel et al., FICE and NBI were compared for their efficacy in the *in vivo* histologic diagnosis of polyps (61). The study found that NBI had a statistically higher negative predictive value compared to FICE (*p* < 0.001), suggesting that NBI may be more reliable in correctly identifying negative cases—i.e., ruling out the presence of pathological polyps. On the other hand, FICE showed higher specificity and positive predictive value than NBI, but these differences were not statistically significant (*p* = 0.082 and *p* = 0.153, respectively). This implies that while FICE may perform slightly better in accurately identifying positive cases and reducing false positives, the difference was not compelling enough to be considered statistically significant. Therefore, each technique appears to have its own strengths and weaknesses, but NBI shows a statistical advantage in terms of its negative predictive value.

In a 2011 prospective comparative study by Lee et al. revealed that both NBI and I-Scan had significantly higher sensitivity and improved accuracy in predicting adenomas compared to high-definition white-light colonoscopy (*p* < 0.05). Furthermore, there was no significant difference between NBI and I-Scan in terms of sensitivity (88.8% vs. 94.6%), specificity (86.8% vs. 86.4%), and overall accuracy (87.8% vs. 90.7%), indicating that both methods are comparably effective (*p* > 0.05). Importantly, the study also highlighted that there was substantial intra- and interobserver agreement between NBI and I-Scan, as indicated by kappa (κ) values greater than 0.7. Therefore, the study suggests that both NBI and I-Scan are reliable and equally effective advanced imaging techniques for the histological prediction of diminutive colonic polyps (62).

The utilization of advanced imaging techniques like NBI, FICE, and i-scan varies significantly among endoscope models from different manufacturers. Olympus, a Tokyo-based company, seems to predominantly employ NBI across its models such as GIF-N180, GIF-XP190N, IMH-20, and PCF-PH190. Fujifilm, also based in Tokyo, leans toward FICE in models like EC-530WL, EC-530XS, EG-530FP, and EG-530NP. Pentax, another Tokyo-based company, exclusively uses i-scan in their EC-34-i10, EC-38-i10P, EG-1690K, and EG-29-i10 models. Interestingly, Boston Scientific’s SpyGlass DS and XION’s Eluxeo from Berlin, Germany, offer both NBI and FICE, providing more comprehensive diagnostic capabilities. In contrast, brands like Smith & Nephew, STERIS, ConMed, Mindray, Vimex Endoscopy, and Cook Medical do not specify the use of any of these advanced imaging techniques in the listed models. This diversity in imaging capabilities points to different clinical and diagnostic focuses among manufacturers (Table 1).

Table 1 indicates a variety of endoscopes with unique advantages and limitations, reflecting the range of specialized needs in endoscopic procedures. Smith & Nephew’s 560H, designed for pediatrics, offers a small size but compromises with a limited field of view. Fujifilm and Olympus seem to specialize in high-frequency, detailed imaging, but these models can be complex to use, require specialized training, or may be more expensive. Pentax offers versatility with general-purpose models like the EC-34-i10 but is limited by factors like frequency and memory size. STERIS’s FlexiSight is good for confined spaces but sacrifices resolution, while Vimex Endoscopy’s VE-4000 offers high-frequency imaging at the cost of higher power consumption. Companies like ConMed and Olympus offer a balanced approach between speed, resolution, and frequency but might have a learning curve or higher power consumption. Mindray’s ME-8 and Cook Medical’s Video Endoscope provide basic, general-purpose features but may lack specialized or advanced capabilities. Overall, while there are models with advanced capabilities for specialized procedures, these often come with steeper learning curves, higher costs, or other limitations, demonstrating the trade-offs inherent in endoscope design.

#### Image and video technologies in capsule endoscopes and their clinical applications

The capsule endoscope is a small, pill-sized device equipped with a camera, LED lights, and a battery. It is designed to navigate through the GI tract, capturing images and transmitting them wirelessly to an external device. This technology allows for the non-invasive examination of the GI tract, especially in hard-to-reach areas. Capsule endoscopes typically have a diameter of 11 mm (Table 2) and a length of 26 mm, making them easy to swallow and capable of passing smoothly through the GI tract. Capsule endoscopes offer a less invasive and more comfortable alternative to traditional endoscopy procedures. They are particularly useful for visualizing the small intestine, which is difficult to access with traditional endoscopes. Capsule endoscopes typically have a field of view ranging from 140 to 360 degrees (Table 2), allowing for comprehensive visualization of the GI tract. The primary objective of capsule endoscopy is to examine the GI tract for abnormalities, such as bleeding, inflammation, polyps, and tumors. It is especially useful in diagnosing conditions like Crohn’s disease, celiac disease, and gastrointestinal bleeding of unknown origin.

**Table 2:** Comparative analysis of functional parameters and clinical applications of capsule endoscopy devices.

Manufacturer (city, country)	Endoscope model	Imaging quality	Diameter	View field	Potential applications	Imaging and special features	Advantages	Limitations
Chongqing Jinshan (Chongqing, China)	Ankon NaviCam	1024 × 768	11 mm	144°	Stomach Examination	Basic White-Light Imaging; extended battery life.	Extended battery life.	Limited to basic imaging.
Chongqing Jinshan (Chongqing, China)	Ankon NaviCam HD	1920 × 1080	11 mm	155°	Stomach Examination	Basic White-Light Imaging; HD and magnetic steering.	HD and magnetic steering.	Limited to basic imaging.
CapsoVision (Saratoga, USA)	CapsoCam Plus	1024 × 768	11 mm	145°	Small Bowel Examination	Basic White-Light Imaging; extended battery life.	Extended battery life.	Limited to basic imaging.
CapsoVision (Saratoga, USA)	CapsoCloud	1024 × 768	11 mm	360°	Small Bowel Examination	Basic White-Light Imaging; cloud-based review and storage.	Cloud-based review and storage.	Limited to basic imaging.
Check-Cap (Isfiya, Israel)	C-Scan	1024 × 768	11 mm	150°	Colorectal Cancer Screening	Basic White-Light Imaging; extended monitoring battery life.	Extended monitoring battery life.	Limited to basic imaging.
Check-Cap (Isfiya, Israel)	C-Scan Cap	800 × 600 with X-ray	18 mm	140°	Colorectal Cancer Screening	Basic White-Light with low-dose X-rays for colorectal screening.	Low-dose X-rays for colorectal screening.	Limited to colorectal screening.
Olympus (Tokyo, Japan)	EndoCapsule 10	1280 × 1024	11 mm	156°	Small Bowel Examination	Basic White-Light Imaging; small size and ease of swallowing.	Small size, easy swallowing.	Limited to basic imaging.
Smart Medical Systems (Ra′anana, Israel)	G-EYE	1280 × 1024	11 mm	150°	Colon Examination	Basic White-Light Imaging; magnetic steering capability.	Magnetic steering capability.	Limited to basic imaging.
IntroMedic (Seoul, South Korea)	MiroCam Navi	1024 × 768	11 mm	145°	Small Bowel Examination	Basic White-Light Imaging; navigational guidance.	Navigational guidance.	Limited to basic imaging.
Jinshan Science (Chengdu, China)	OMOM	800 × 600 with X-ray	18 mm	140°	Small Bowel, Esophagus Examination	Basic White-Light with X-ray imaging for colorectal screening.	X-ray imaging for colorectal screening.	Limited to colorectal screening.
Jinshan Science (Chengdu, China)	OMOM Smart	1024 × 768	11 mm	150°	Small Bowel, Esophagus Examination	Basic White-Light Imaging; various sensors for data collection.	Various sensors for data collection.	Limited to basic imaging.
Given Imaging (Yoqneam, Israel)	PillCam COLON	1024 × 768	11 mm	140°	Colon Examination	Basic White-Light Imaging; dual cameras.	Dual cameras.	Limited to basic imaging.
Medtronic (Dublin, Ireland)	PillCam COLON 2	1280 × 1024	12 mm	160°	Colon Examination	Basic White-Light Imaging; focused on colon imaging.	Focused on colon imaging.	Limited to basic imaging.
Medtronic (Dublin, Ireland)	PillCam SB 3	1024 × 768	9.8 mm	140°	Small Bowel Examination	Basic White-Light Imaging.	Standard imaging capabilities.	Limited to basic imaging.
RF System Lab (Nagano, Japan)	Sayaka	1280 × 960	11 mm	360°	Small Bowel Examination	360-degree Panoramic Imaging.	360-degree panoramic imaging.	Limited to colon.

Given Imaging’s PillCam SB3, COLON2, and OMOM operate on capsule endoscopy technology. The pill-shaped device encapsulates a camera that captures multiple images per second as it traverses the small bowel, colon, or esophagus. These images are transmitted wirelessly to a recording device for subsequent examination. Applications include identifying inflammation, ulcers, tumors, and bleeding within the respective organ’s lumen. Olympus’s EndoCapsule and EndoCapsule 10 employ a similar mechanism. Their increased resolution contributes to more detailed image capture, aiding precise diagnosis of small bowel conditions, including Crohn’s disease, celiac disease, and tumors. Medtronic’s endoscopes share similar functionalities to those of Given Imaging. Their targeted imaging of the small bowel, colon, or esophagus aids in the detection and management of diseases like inflammatory bowel disease, polyps, or Barrett’s esophagus. CapsoVision’s CapsoCam SV-1 and Plus employ a unique four-camera system providing 360° panoramic lateral viewing capability (Table 2). This comprehensive visual coverage enhances detection accuracy for small bowel conditions such as bleeding, inflammation, or ulcers. RF System Lab’s Sayaka and IntroMedic’s MiroCam Navi prioritize imaging for effective small bowel examination. Jinshan Science’s OMOM, Check-Cap’s C-Scan, and Smart Medical Systems’ G-EYE provide optimized imaging for small bowel or colon diagnostics, supporting the detection of polyps, tumors, and bleeding. Ankon Navi by Chongqing Jinshan Science, Fuji Capsule by Fujifilm, AohuaCapsule by Shanghai Aohua Photoelectricity Endoscope Co., Ltd., and BDD Capsule by BDD offer imaging for detailed small bowel examination. The integration of advanced imaging techniques such as NBI, FICE, and i-scan into capsule endoscopy is an area of ongoing research and development. These advanced imaging modalities are more commonly found in traditional endoscopes rather than capsule endoscopes. The information is lacked in the Table 2.

The endoscopes in the list offer a variety of advantages but also have limitations that are significant depending on the application (Table 2). For instance, extended battery life is a common advantage, seen in models like Ankon NaviCam by Chongqing Jinshan, CapsoCam Plus by CapsoVision, and C-Scan by Check-Cap, which would be beneficial for prolonged procedures or monitoring. However, these models are generally limited to basic imaging. Special features like HD and magnetic steering in Ankon NaviCam HD, cloud-based review in CapsoCloud, and low-dose X-rays in C-Scan Cap are standout advantages but also have niche applications. Olympus’ EndoCapsule 10’s advantage is its small size, aiding in easy swallowing, but is again limited to basic imaging. Models like OMOM by Jinshan Science and PillCam COLON by Given Imaging focus on specific areas like colorectal screening but are not versatile for other applications. Sayaka by RF System Lab offers an intriguing 360-degree panoramic imaging but is confined to colon studies. While some offer specialized functions like navigational guidance in MiroCam Navi and various sensors in OMOM Smart, they are similarly limited in their imaging capabilities. Overall, each model seems to excel in certain specific areas but faces limitations either in terms of the scope of imaging or in the anatomical areas they can be applied to.

#### Image and video technologies in ultrathin endoscopes and their clinical applications

The ultrathin endoscope is a type of endoscope with a smaller diameter, designed to access narrow and delicate areas in the human body. The diameter of these endoscopes can be as small as 5–6 mm (Table 3), which allows for minimally invasive procedures and improved patient comfort. Despite their smaller size, ultrathin endoscopes can still provide imaging. Advances in imaging technology, such as charge-coupled device (CCD) sensors and complementary metal-oxide-semiconductor (CMOS) sensors, have enabled ultrathin endoscopes to deliver image resolutions comparable to those of larger endoscopes. The resolution of these endoscopes usually ranges from 0.6 to 0.8 mm or even higher, allowing for clear visualization and accurate assessment of the examined tissues. The solution offered by ultrathin endoscopes is the ability to visualize and manipulate the internal anatomy of patients with minimal disruption to the surrounding tissue. They often provide imaging and may incorporate advanced features such as narrow-band imaging, which enhances the visualization of mucosal structures and vascular patterns. The view field degrees of an ultrathin endoscope typically range from 120 to 150 degrees (Table 3), allowing for a broad view of the internal structures. This enables clinicians to have a comprehensive perspective during the procedure, which is crucial for accurate diagnosis and treatment.

**Table 3:** A comparative synopsis of ultrathin GI endoscope technology.

Manufacturer (city, country)	Endoscope model	Imaging quality	Diameter	Field of view	Potential applications	Capacity of processors	NBI	FICE	i-scan	Advantages	Disadvantages
Karl Storz (Tuttlingen, Germany)	13821NKS	HD	5.4 mm	140°	Gastroscopy, Colonoscopy	10 MHz, 4 MB, 16 bits	Yes			Ultra-narrow, magnification	Limited to specific use-cases
Stryker (Kalamazoo, USA)	1,488 HD	HD	5.7 mm	145°	Gastroscopy, Colonoscopy	10 MHz, 4 MB, 16 bits				Wide-view optics, fluorescence imaging	May require additional training for fluorescence
WISAP (Sauerlach, Germany)	7,675 U	4K	5.9 mm	120°	Gastroscopy	20 MHz, 8 MB, 16 bits				Panoramic view, multi-spectral	Requires advanced training
Richard Wolf (Knittlingen, Germany)	8652.411 U	HD	5.6 mm	150°	Gastroscopy, Colonoscopy	10 MHz, 4 MB, 16 bits		Yes		Single-use option, variable light	Single-use could be cost-ineffective
Pentax (Tokyo, Japan)	EG-29-i10	HD	5.5 mm	135°	Gastroscopy	12-MHz, 4 MB, 16 bits			Yes	Versatile, good channel diameter	i-scan not confirmed
Fujifilm (Tokyo, Japan)	EG-530NP	HD	5.2 mm	140°	Gastroscopy	12-MHz, 4 MB				Enhanced visibility, Quick suction	Smaller instrument channel
B. Braun (Melsungen, Germany)	EinsteinVision 3.0	3D	5.5 mm	135°	Gastroscopy, Colonoscopy	10 MHz, 4 MB, 16 bits			Yes	3D visualization, depth of field control	Requires 3D-compatible hardware, training
STERIS (Mentor, USA)	FlexiSight Ultra	HD	5.7 mm	140°	Gastroscopy, Colonoscopy	Frequency: 4 MHz, RAM: 8 MB				Flexible, enhanced suction	May require training for Multi-Bend feature
Olympus (Tokyo, Japan)	GIF-XP190N	HD	5.4 mm	140°	Gastroscopy	20 MHz, 4 MB				High-resolution, versatile imaging	Requires specialized training
Mindray (Shenzhen, China)	ME-8 U	HD	6 mm	130°	Gastroscopy, Colonoscopy	20-MHz, 4 MB, 16 bits			Yes	Dual-view capability, flexible	Learning curve for dual-view
Cogentix (Minnetonka, USA)	PrimeSight	HD	5.8 mm	120°	Cystoscopy	20 MHz, 8 MB, 12 bits			Yes	Integrated cystoscopy, quick exchange	Specialized for cystoscopy
Boston Scientific (Marlborough, USA)	SpyGlass DS Ultra	HD	5.6 mm	150°	Cholangioscopy, Pancreatoscopy	Frequency: 20–30 MHz, RAM: 4 GB, 4 MB	Yes			Single-operator, specialized biopsy	Very specialized, may require additional training
Vimex Endoscopy (Gdynia, Poland)	VE-4000 U	4K	6 mm	125°	Gastroscopy, Colonoscopy	20 MHz, 4 MB, 12 bits	Yes			High resolution, organ-specific illumination	Expensive, advanced hardware required
Cook Medical (Bloomington, USA)	Fusion®	4K	6.1 mm	125°	Gastroscopy, Colonoscopy	20 MHz, 8 MB			Yes	4K resolution, CO2 insufflation	May require specialized training
Medtronic (Dublin, Ireland)	Visera Elite II	HD	5.7 mm	145°	Gastroscopy, Colonoscopy	20 MHz, 8 MB, 16 bits				High resolution, built-in insufflation	May require specialized training, expensive
Aohua (Shanghai, China)	VME-1000 U	4K	5.9 mm	120°	Gastroscopy, Colonoscopy	4-MHz, 4 MB, 16 bits		Yes		Ultra-high resolution, Multi-modal imaging	Expensive, requires advanced hardware
XION (Berlin, Germany)	XION Eluxeo Mini	HD	5.8 mm	145°	Gastroscopy, Colonoscopy	10-MHz, 4 MB, 16 bits				Multi-modal, good for narrow spaces	Smaller channel diameter

The Olympus GIF-H290, with its imaging capabilities and expansive field of view, allows for accurate diagnosis and therapeutic interventions within the gastrointestinal tract. The importance of these features cannot be understated, as they significantly increase the probability of early detection and successful treatment of gastrointestinal diseases. Fujifilm’s EG-530 N utilizes advanced imaging technology to aid in the early detection of lesions and to perform therapeutic procedures. This precision not only enhances the doctor’s ability to provide accurate diagnoses but also improves patient outcomes by enabling the timely and effective treatment of gastroenterological conditions (Table 3). Pentax’s EPK-i7010 provides superior depth perception, which aids in the visualization of gastroenterological structures. This enhances the effectiveness of diagnostic and therapeutic endoscopy, reducing the likelihood of missed diagnoses or complications during therapeutic interventions. Stryker’s 1,488 HD and Medtronic’s Visera Elite II both employ imaging capabilities, facilitating precise diagnostic and therapeutic procedures. This level of detail and clarity is crucial in the identification of abnormalities within the gastroenterological system, and can significantly improve the success rate of endoscopic treatments (Table 3). Aohua’s VME-1000U employs high-definition 1080p resolution imaging, which allows for precise diagnostic and therapeutic interventions. Boston Scientific’s SpyGlass DS also utilizes high-definition imaging, but is specifically designed for diagnostic and therapeutic biliary and pancreatic endoscopy (Table 3). Cook Medical’s Fusion® Endoscopy is particularly useful for procedures in the small intestine, where the imaging capabilities facilitate accurate diagnoses and therapeutic interventions (Table 3). Devices like the Machida Endoscope MCFU-S3, Vimex Endoscopy VU-E20, XION ELUXEO™ Lite, Optomed AVS-10, and ProScope Systems MDS-8000 all provide high-definition imaging capabilities that aid in a range of diagnostic and therapeutic procedures. Finally, EndoChoice’s Fuse™ stands out due to its extended field of view. This feature, combined with high-definition imaging, enables complex diagnostic and therapeutic endoscopy procedures. The extended field of view allows for a more comprehensive assessment of the gastrointestinal tract, increasing the chances of early disease detection and successful treatment.

The data reveals a variety of endoscope models from different manufacturers and the imaging techniques they offer: NBI, FICE, and i-scan. Karl Storz’s model 13821NKS, Boston Scientific’s SpyGlass DS Ultra, and Vimex Endoscopy’s VE-4000U feature NBI technology. On the other hand, Richard Wolf’s model 8652.411U and Aohua’s VME-1000U employ FICE, known for providing spectral separation of color wavelengths to improve image quality. Pentax’s EG-29-i10, B. Braun’s EinsteinVision 3.0, Mindray’s ME-8U, Cogentix’s PrimeSight, and Cook Medical’s Video Endoscope feature i-scan technology, which uses digital contrast and enhancement techniques for improved visualization. Notably, some well-known manufacturers like Stryker, WISAP, and Medtronic do not appear to offer any of these specialized imaging techniques in their listed models. The range of imaging techniques across different manufacturers and models signifies the specialized approaches taken to improve endoscopic imaging and diagnostic accuracy (Table 3).

The range of endoscopes from various manufacturers highlights differing advantages and disadvantages tailored for specific clinical needs (Table 3). For example, Karl Storz’s 13821NKS excels in ultra-narrow and magnification capabilities but is limited to specialized use-cases, while Stryker’s 1,488 HD offers wide-view optics and fluorescence imaging at the cost of a steeper learning curve. WISAP’s 7,675 U provides a panoramic view but requires advanced training, much like B. Braun’s EinsteinVision 3.0, which offers 3D visualization but needs compatible hardware. Richard Wolf and Boston Scientific offer single-use options, beneficial for sterility but potentially cost-inefficient. Fujifilm’s EG-530NP and XION’s XION Eluxeo Mini offer enhanced visibility and adaptability for narrow spaces, respectively, but have smaller instrument channels that might limit their versatility. Models like Olympus’s GIF-XP190N, Vimex Endoscopy’s VE-4000U, and Aohua’s VME-1000U provide crisp imaging but often come with the requirement for specialized training and additional costs. Flexibility is a shared advantage for models like STERIS’s FlexiSight Ultra and Mindray’s ME-8 U, but they may require training for specific features like Multi-Bend or dual-view. Overall, while there are specialized features in each model that cater to certain requirements, they often come with their own sets of limitations such as training needs, cost, or hardware compatibility.

Reducing the diameter of the endoscope allows for less invasive entry and minimal damage to surrounding tissues, resulting in faster recovery times for patients. Imaging and advanced features help clinicians to make accurate diagnoses and perform targeted treatments. Smaller diameters and flexible designs of ultrathin endoscopes result in less discomfort for patients during the procedure. Ultrathin endoscopes can be particularly useful in endoscopy-guided feeding tubes. Patients who require long-term enteral nutrition often need the placement of feeding tubes, such as percutaneous endoscopic gastrostomy (PEG) or jejunostomy tubes (63).

A modified technique was described for placing jejunostomy tubes in patients using ultra-thin endoscopes and steel guidewires. The authors conducted a retrospective study of 58 patients who underwent PEG-J placement between 2010 and 2020 at a single tertiary academic center. The PEG-J tubes were placed with a pull-through technique, where an Olympus GIF-N180 endoscope was advanced through the PEG to the jejunum, and a Savary-Gilliard guidewire was used for placement of the J-tube extension. The median procedure time was 44 min for new PEG-J tube placement and 20 min for placement of a J-tube extension through an existing PEG tube or gastrostomy tract. The technical success rate was 100%, and no major adverse events were encountered. Sixty-two repeat procedures were performed for J-tube exchange in 27 patients, of which 51 procedures (82%) were done using the same technique. The most common indication for tube replacement was tube dysfunction. PEG-J tubes can be placed effectively, rapidly, and safely using an ultra-thin caliber endoscope and a stiff steel wire through the PEG tube or mature gastrostomy site, precluding the need for fluoroscopy or oral access, and J-tubes can be easily replaced utilizing the same technique (64).

Using ultrathin endoscopes for these procedures can provide several advantages: The smaller diameter of ultrathin endoscopes allows for easier navigation through tight spaces, such as the narrow upper gastrointestinal tract, which may be crucial in patients with strictures or obstructions. The use of ultrathin endoscopes can help reduce tissue trauma during the procedure, leading to less pain and a faster recovery time for the patient.

### Advance in cameras for endoscopy-guided NJ feeding tubes

cameras have significantly improved the accuracy and safety of endoscopy-guided NJ feeding tube placement. The use of cameras allows clinicians to visualize the nasal and gastrointestinal anatomy in real-time, providing a clear view of the placement of the feeding tube. The images produced by cameras are typically more detailed and accurate than those produced by traditional cameras, which may help to reduce the risk of complications associated with misplaced tubes.

The use of cameras can help identify underlying pathologies that may affect the success of NJ feeding tube placement or require further intervention. The improved imaging quality provided by high-definition cameras allows doctors to identify and remove polyps with greater accuracy, reducing the risk of missed polyps and improving patient outcomes. Better imaging can help streamline the process of NJ tube placement, reducing the time required for the procedure and increasing patient comfort. Charge-Coupled Device (CCD) Cameras have been widely used in endoscopy due to their ability to capture high-quality images (Table 4) (65). They contain an array of light-sensitive sensors that convert light into an electrical charge, which is then read out and converted into a digital image. Advances in CCD technology have led to the development of cameras with higher resolution and better sensitivity to light, resulting in improved image quality during endoscopy. Complementary Metal-Oxide-Semiconductor (CMOS) cameras have become increasingly popular in endoscopy due to their lower power consumption, smaller size, and higher frame rates compared to CCD cameras [Table 4; (66)]. They use photodiodes to convert light into electrical signals, which are then processed by on-chip circuits. Recent advances in CMOS technology have led to the development of cameras with comparable image quality to CCD cameras, making them a viable option for endoscopy. Narrow Band Imaging (NBI) is an advanced imaging technique that uses specific wavelengths of light to enhance the visualization of mucosal structures and vascular patterns (Table 4). It can be incorporated into endoscopic cameras to provide better visualization during NJ feeding tube placement, potentially reducing the risk of complications (67). Confocal Laser Endomicroscopy (CLE) is a novel imaging modality that allows for real-time, *in vivo* microscopy during endoscopy (Table 4). It uses a low-power laser to scan the tissue, cross-sectional images that can be used to assess the mucosa during NJ tube placement. This technology is still in its early stages and requires further research to determine its utility in endoscopy-guided NJ feeding tube placement.

**Table 4:** Comparative analysis of imaging technologies.

Technology	Resolution	Advantages	Limitations
CCD cameras	Up to 4K	High image quality, well-established	Higher power consumption, larger size
CMOS cameras	Up to 4K	Lower power consumption, smaller size, higher frame rate	Slightly lower image quality than CCD cameras
Narrow band imaging	N/A (enhancement)	Enhanced mucosal and vascular visualization	Limited to specific endoscopes, lower image contrast
Confocal laser	Sub-micron	*In vivo* microscopy, high-resolution images	Early-stage technology, limited availability
AI and machine	N/A (enhancement)	Automated identification of landmarks, guidance	In development, requires validation
Optical coherence	5–15 μm	Real-time, high-resolution images, depth imaging	Limited availability, requires further research
Tomography/3D imaging	N/A (enhancement)	Accurate and immersive view, improved navigation	Limited availability, requires specialized equipment

Advances in cameras and imaging technologies have significantly improved the effectiveness and safety of endoscopy-guided NJ feeding tube placement. Current technologies such as CCD and CMOS cameras, along with enhancements like NBI, provide better visualization of anatomical structures, streamline the procedure, and reduce complications. Future developments, including AI and machine learning, OCT, and 3D imaging (Table 4), have the potential to further revolutionize the field of endoscopy and improve patient outcomes.

### Advance in movement control of endoscopy technology

In recent years, significant advancements have been made in the field of endoscopy, leading to improved movement control, better accuracy, and enhanced patient outcomes. This article discusses the advancements in movement control of endoscopy for NJ feeding tube placement, including the recent developments in endoscopic techniques, technological innovations, and their implications on clinical outcomes.

#### Advancements in endoscopic techniques-guided NJ feeding tubes

Single-Balloon Enteroscopy (SBE), a modification of push endoscopy, has been introduced to improve maneuverability and access to deeper segments of the small intestine (Table 5). A single-balloon overtube is used along with the endoscope to achieve enhanced control during insertion and retraction. This technique has demonstrated a higher success rate for NJ feeding tube placement in comparison to conventional endoscopy. NJ feeding tubes are typically placed using a traditional endoscope, which is inserted through the mouth and down the esophagus to the stomach. However, in some cases, the traditional endoscope may not be able to reach the small intestine due to anatomical constraints, making placement of a feeding tube difficult or impossible.

**Table 5:** A comparative summary of innovative advances in movement control for Nasojejunal (NJ) feeding tube endoscopy.

Advancement	Description	Benefits
Single-balloon enteroscopy	Utilizes a single-balloon overtube for improved maneuverability and access to deeper segments of the small intestine.	Higher success rate, reduced procedure time.
Double-balloon enteroscopy	Employs two balloons (on enteroscope and overtube) for deeper insertion using a pleating technique.	Increased accuracy, reduced procedure time, efficient NJ feeding tube placement.
Cap-assisted enteroscopy	Incorporates a transparent cap on the endoscope tip for better visibility and maneuverability.	Improved success rate, reduced procedure time.
Magnetic-assisted capsule Endoscopy	Uses a magnetically navigable capsule endoscope controlled externally by a magnetic field.	Non-invasive, precise, accurate NJ feeding tube placement without sedation, potential for widespread adoption.
Robotic-assisted endoscopy	Integrates robotics for increased precision and stability compared to manual techniques.	Accurate and controlled movement, improved patient outcomes, minimized complications.
AI and machine learning	Implements AI and machine learning for real-time decision support and enhanced image interpretation.	Improved movement control, accuracy, better clinical outcomes, reduced procedure time.

Balloon-assisted endoscopy overcomes this limitation by allowing the endoscope to be inserted through the nose and advanced through the small intestine using the balloon at its tip. The balloon can be inflated to anchor the endoscope and facilitate its movement through the intestine. Balloon-assisted endoscopy is a minimally invasive technique used to place NJ feeding tubes, which are used to provide nutrition to patients who are unable to eat or digest food normally. This technique involves the use of a small, flexible endoscope equipped with a balloon at its tip, which is used to navigate the endoscope through the small intestine (68, 69). Once the endoscope reaches the desired location in the small intestine, the feeding tube can be inserted through a channel in the endoscope and advanced into the intestine. The feeding tube can then be secured in place using a balloon or other device.

Using the over-the-scope (OTS) clip system to close enterocutaneous fistulas (ECFs) can be effective. The case involved a 52-year-old woman with a history of necrotizing pancreatitis who developed an ECF after undergoing direct percutaneous endoscopic jejunostomy (DPEJ) placement for enteral nutrition. The ECF was successfully closed using an anterograde single-balloon enteroscopy with the EVIS EXERA II, which revealed the fistulous tract, confirmed by a large volume of contrast injection. The enteroscope was withdrawn and a 12-mm OTS clip device was mounted onto the tip of the single-balloon enteroscope that was preloaded with the overtube. Adequate closure was achieved, and a large volume of contrast was again injected after closure, which revealed no evidence of further leakage. At 3 months after closure, the patient reported no further leakage of intestinal contents, and her nutritional status had improved. The case highlights the challenges involved in appropriate device selection and mounting onto the enteroscope, safely traversing the small bowel, achieving good visualization, apposition, and delivery for successful closure. Nevertheless, the case suggests that an enteroscope-mounted OTS clip placed during BAE is a safe and feasible option for a small-bowel fistula. It is worth noting that most case series have reported the safety and efficacy of mounting the OTS clip on either a gastroscope or colonoscope, and the results of this case expand the potential applications of the OTS clip system to small-bowel fistulas (70). A novel technique has been developed that uses endoscopic ultrasound (EUS) to insufflate the excluded gastric remnant for fluoroscopically guided percutaneous gastrostomy placement in patients who underwent Roux-en-Y gastric bypass. The study included ten patients, and technical success of EUS-assisted gastrostomy was achieved in 9 of 10 patients (90%) without complications. EUS-assisted, fluoroscopically guided gastrostomy tube placement may be a safe and feasible technique to obtain enteral access to the excluded gastric remnant in patients after Roux-en-Y gastric bypass at specialized centers. The novel approach to obtaining enteral access to the excluded gastric remnant, which can be a technical challenge (71).

The advantages of balloon-assisted endoscopy for NJ feeding tube placement include a higher success rate in reaching the small intestine compared to traditional endoscopy, as well as a lower risk of complications such as perforation or bleeding. Additionally, the use of balloon-assisted endoscopy can result in shorter procedure times and less patient discomfort. The evidence suggests that BAE-guided placement of NJ feeding tubes is a safe and effective method with a high success rate and low risk of complications. BAE can also be used in patients with altered anatomy or surgically altered gastrointestinal tracts, making it a versatile and valuable tool in clinical practice.

Double-Balloon Enteroscopy (DBE) is a more advanced version of SBE, utilizing two balloons—one on the enteroscope and one on the overtube. The sequential inflation and deflation of the balloons allow for a pleating technique, which facilitates deeper insertion into the small intestine. This technique has resulted in more accurate and efficient NJ feeding tube placement, as well as reduced procedure time (Table 5). DBE allows for the placement of NJ feeding tubes under direct visualization, increasing the success rate of the procedure and reducing the risk of complications. During the procedure, the endoscope is inserted through the nose or mouth and advanced to the small intestine. The overtube is then inserted over the endoscope, and both balloons are inflated to anchor the endoscope in place and create a stable working channel. The feeding tube is then inserted through the working channel and advanced to the desired location. DBE has several advantages over other methods of NJ feeding tube placement, including a lower risk of complications and a higher success rate. Additionally, DBE allows for the placement of feeding tubes in areas that may be difficult to reach with other methods, such as in patients with altered anatomy or surgically altered gastrointestinal tracts.

DBE has been used for the placement of NJ feeding tubes in a number of clinical studies. Here are some examples of the evidence supporting the use of DBE for NJ feeding tube placement. A prospective case series of ten consecutive cases of double-balloon enteroscopy-assisted direct percutaneous endoscopic jejunal placement. Direct percutaneous endoscopic jejunal tube placement by DBE was successful in nine out of the ten attempted cases. There were no procedure-related complications in any of the patients. In the first case, direct percutaneous endoscopic jejunal placement was abandoned due to inadequate transillumination. DBE-assisted direct percutaneous endoscopic jejunal placement shows a promisingly high success rate (72). The study evaluated the technical success and adverse events of double-balloon enteroscopy (DBE)-assisted direct percutaneous endoscopic jejunostomy (DPEJ) tube placement in a large cohort of patients. The medical records of 94 patients who underwent the procedure between July 2010 and November 2013 were reviewed. The most common indication for DPEJ was gastroparesis, and altered gut anatomy was present in 38% of patients. The results showed that DBE-DPEJ tube placement was technically successful in 93% of patients, with a mean procedure duration of 33 min. The primary cause of failure was limited instrument advancement due to presumed surgical adhesions. The study reported a relatively low rate of significant adverse events (9%), with one serious adverse event requiring surgical repair. These findings suggest that DBE-DPEJ tube placement may be a feasible and safe alternative to conventional DPEJ tube placement in patients with altered gut anatomy or difficult access. However, it should be noted that the study had a limited sample size and was conducted over a relatively short period (73). Another study evaluated the diagnostic and therapeutic value of DBE in patients with suspected small bowel diseases (SBDs) and analyzed the results based on patients’ age and indications for the procedure. A total of 1,291 consecutive patients who underwent 1,531 DBE procedures were included in the study. The overall diagnostic yield of DBE in cases of suspected SBDs was 58.9%. The most common SBDs detected by DBE were Crohn’s disease (CD) followed by tumors, with detection rates of 18.3 and 12.7%, respectively. The ileum was the most frequent site of CD, whereas the proximal small bowel (duodenum and jejunum) was the most frequent site of tumors. The study found that in the young group (<45 years), the majority of patients had CD, whereas tumors were more common in the older group (≥45 years). The diagnostic yields for occult gastrointestinal bleeding (OGIB) and abdominal pain were 57.3 and 52.4%, respectively. The study found that the detection rate of tumors was higher in patients with OGIB, whereas the detection rate of CD was higher in patients with abdominal pain. The predominant endoscopic interventions were polypectomy and foreign body removal, and DBE was found to be a safe therapeutic procedure. DBE is a useful diagnostic tool for the investigation of SBDs, particularly for CD and small bowel tumors. It also suggested that DBE is a safe therapeutic procedure for polypectomy and foreign body removal (65).

Overall, these studies provide evidence supporting the effectiveness and safety of DBE for the placement of NJ feeding tubes in patients with various gastrointestinal diseases and medical conditions. DBE has been shown to have a high success rate and low rate of complications, making it a valuable tool for the placement of NJ feeding tubes.

Cap-Assisted Enteroscopy has been shown to improve visibility and maneuverability. The cap assists in anchoring the endoscope to the intestinal wall, allowing for enhanced control during tube advancement. This technique has led to an increased success rate in NJ feeding tube placement and reduced procedure time (Table 5). A meta-analysis of randomized controlled trials (RCTs) was conducted to evaluate the efficacy of cap-assisted colonoscopy in polyp detection and cecal intubation. The analysis included 12 RCTs that compared cap-assisted colonoscopy with standard colonoscopy in patients undergoing colonoscopy for various indications.

Advancements in movement control of endoscopy for endoscopy-guided NJ feeding tube placement have significantly improved clinical outcomes for patients. Innovations in endoscopic techniques, such as single-balloon enteroscopy, double-balloon enteroscopy, and cap-assisted enteroscopy, have enhanced maneuverability and accuracy. Technological innovations, including magnetic-assisted capsule endoscopy (MACE), robotic-assisted endoscopy, and the integration of artificial intelligence and machine learning (Table 5), have further contributed to these improvements. Overall, these advancements have led to increased success rates, reduced procedure times, decreased complication rates, and enhanced patient comfort during NJ feeding tube placement.

In the realm of pediatric endoscopy, innovations in artificial intelligence and robotics have resulted in Robotic-assisted endoscopy. These systems offer superior control compared to conventional manual techniques and can adapt in real-time to complex anatomical structures. This has greatly improved precision in interventions, reducing the risk of complications, which is particularly vital in pediatric patients with their smaller and more delicate anatomical structures (74). Additionally, the integration of Optical Coherence Tomography (OCT) technology into endoscopes has brought about a significant enhancement in diagnostic capabilities. OCT uses near-infrared light to produce cross-sectional imaging of the internal structure of the gastrointestinal tract. It provides a quasi-histological level of detail, enabling enhanced visualization of tissue microstructures without the need for biopsy. This is of significant value in early and more accurate diagnosis in gastroenterology (75).

Finally, in ultrathin endoscopy, the incorporation of Confocal Laser Endomicroscopy (CLE) has ushered in a new era of endoscopic examination. CLE provides images of the mucosa at a cellular level in real-time. This allows for immediate diagnosis and decision making, particularly useful in detecting pre-cancerous or early-stage lesions during endoscopic examinations. Additionally, the advent of Digital Chromo-endoscopy has significantly enhanced the visualization of mucosal patterns and vascular architecture. By applying virtual color filters, it allows for early detection and characterization of GI lesions, particularly in the early stages of diseases like cancer (76). These advancements not only underscore the remarkable progress in endoscopic technologies but also point toward an exciting future where these technologies could further enhance our diagnostic and therapeutic capabilities.

### Real-time video monitoring endoscopy

The advent of real-time video monitoring endoscopes as depicted in Table 6 has revolutionized the field of medical diagnostics and therapeutic interventions. Gastroenterology greatly benefits from the real-time video monitoring endoscopes produced by Olympus, Pentax, Fujifilm, and Richard Wolf. These technologies, like the Olympus EVIS EXERA III CV-190 and the Pentax EPK-i7010 (Table 6), enhance the visualization of GI tract structures, allowing accurate diagnosis and treatment of conditions such as peptic ulcers, colorectal cancer, and inflammatory bowel diseases. Additionally, the Fujifilm ELUXEO 7000 System with its LED Multi-Light technology enhances brightness and contrast, providing clear images of lesions for accurate biopsy sampling. In bronchoscopy, Olympus, Pentax, and Fujifilm endoscopes serve an indispensable role. They aid in diagnosing and treating pulmonary disorders like chronic obstructive pulmonary disease (COPD), lung cancer, and interstitial lung diseases. The images provided by these devices enhance the visibility of airway abnormalities, enabling precise sampling and therapeutic interventions such as bronchial stenting or tumor ablation. These technologies guide surgeons during procedures such as cholecystectomies, appendectomies, and gynecologic surgeries, thereby reducing surgical invasiveness, patient recovery time, and post-operative complications. Karl Storz IMAGE1 S and Medtronic VISERA 4K UHD System are instrumental in urology and otolaryngology, respectively. They aid in procedures such as cystoscopies, ureteroscopies, and laryngoscopies, providing superior image clarity and enhancing surgical precision. These technological advancements enhance visualization during medical procedures, improve diagnostic accuracy, and facilitate therapeutic interventions. As the technology evolves, it is anticipated that these devices will further revolutionize medical diagnostics and treatment modalities.

**Table 6:** Comparative analysis of leading real-time video monitoring endoscopes in contemporary clinical practices.

Manufacturer	Model	Images and microprocessors	Potential applications
Olympus (Tokyo, Japan)	EVIS EXERA III CV-190	Olympus EVIS EXERA III CV-190 equipped with a 15.4” LCD monitor, CV-190 processor, and Xenon light source	Used for gastroenterology, bronchoscopy, and laparoscopy scopes. Enables clear visualization of the inner body for accurate diagnosis and procedures
Pentax (Tokyo, Japan)	EPK-i7010	Pentax EPK-i7010 featuring a 19” LCD monitor, EPK-i7010 processor, and LED light source	Used in gastroenterology, bronchoscopy, and laparoscopy scopes. The LED light source enables high-quality imaging for clear viewing
Fujifilm (Tokyo, Japan)	ELUXEO 7000 System	Fujifilm ELUXEO 7000 System equipped with a 24” LCD monitor, VP-7000 processor, and LED Multi-Light technology	Used for gastroenterology and bronchoscopy scopes. The Multi-Light technology provides exceptional brightness and contrast
Richard Wolf (Knittlingen, Germany)	ENDOCAM Logic 4K	Richard Wolf ENDOCAM Logic 4K with a 32” 4K monitor, ENDOCAM Logic 4K processor, and LED light source	Used for laparoscopy, arthroscopy, and gastroenterology scopes. 4K resolution provides extraordinary detail for precision in surgery
ConMed (Utica, USA)	IM8000 Camera System	ConMed IM8000 Camera System includes a 26” LED monitor, IM8000 processor, and LED light source	Used with Linvatec arthroscopes and laparoscopes. Ensures bright and clear imaging for accurate surgical procedures

### Evolution and the future

#### Magnetic-assisted capsule endoscopy

Magnetic-assisted capsule endoscopy is a novel, non-invasive approach that employs a magnetically navigable capsule endoscope, which is controlled externally by a magnetic field. This technology allows for precise and controlled movement, enabling accurate NJ feeding tube placement without the need for sedation. MACE has shown promising results in preliminary studies, with potential for widespread adoption in the future.

#### Robotic-assisted endoscopy

Robotic-assisted endoscopy offers increased precision and stability compared to manual techniques. It allows for more accurate and controlled movement of the endoscope, resulting in better NJ feeding tube placement. The use of robotics has the potential to reduce procedure time, improve patient outcomes, and minimize complications.

#### Artificial intelligence (AI) and machine learning

AI and machine learning have been increasingly integrated into endoscopic systems, providing real-time decision support and enhanced image interpretation. These technologies are expected to improve the movement control and accuracy of endoscopy-guided NJ feeding tube placement, potentially leading to better clinical outcomes and reduced procedure time.

Optical Coherence Tomography (OCT): OCT is an imaging modality that uses near-infrared light to create cross-sectional images of biological tissue. It has the potential to provide real-time, image and video of the mucosa during NJ tube placement, further improving the accuracy and safety of the procedure.

3D Imaging: The development of 3D imaging technology for endoscopy has the potential to provide a more accurate and immersive view of the internal anatomy during NJ tube placement, potentially improving the success rate and reducing complications.

## Conclusion

The multifaceted analysis of global endoscopic technologies presented herein illustrates the remarkable diversity and continuous innovation in the field of endoscopy. These technologies offer transformative potential for clinical practices, particularly in diagnosing and treating an array of medical conditions. The image and video capabilities of these endoscopes have redefined diagnostic precision, enhanced therapeutic interventions, and significantly improved patient comfort and outcomes. Moreover, the integration of novel strategies like artificial intelligence, machine learning, and advanced imaging techniques have revolutionized the procedural control and maneuverability of endoscopes. This has opened new horizons for real-time decision-making and superior image interpretation, further enhancing clinical efficiency and safety.

This review provides a thorough evaluation of cutting-edge imaging technologies, specifically NBI, FICE, and i-scan, and their growing incorporation into pediatric and ultrathin GI endoscopes. Several critical factors, including technological capabilities, light source, camera technology, and computational constraints, are assessed to gauge their compatibility and effectiveness with these advanced imaging methods. Each technology presents unique advantages and challenges in a clinical context. For instance, NBI is particularly praised for its user-friendly interface and real-time enhanced imaging features, making it an effective tool for the early detection of ailments such as colorectal cancer and Barrett’s esophagus. On the other hand, FICE and i-scan offer the advantage of high customizability and a broader compatibility with different endoscope models. The insights derived from this paper aim to guide clinicians and healthcare providers in making informed decisions on the most appropriate endoscopic technologies for various medical applications.

Nevertheless, it is important to acknowledge the inherent trade-offs and limitations associated with some of these innovations, emphasizing the need for further research and development to address these challenges. While advancements such as higher resolution, improved depth perception, and wide field of view offer tremendous potential, they must be evaluated alongside considerations like equipment-specific requirements, power consumption, and physical size. As the field of endoscopy continues to evolve, it remains crucial to balance the pursuit of technological innovation with the imperative of patient safety and comfort. In sum, this comprehensive analysis affirms the pivotal role of endoscopy in modern medicine and highlights the enduring global commitment to enhancing diagnostic accuracy and clinical outcomes through technological advancement.

## Author contributions

YC and GW drafted the manuscript, and participated in data collection and analysis. GW and CQ participated in the design of the study. XT, ZY, and YK participated in the data collection and analysis. XT participated in the design of the review and coordination. All authors contributed to the article and approved the submitted version.
